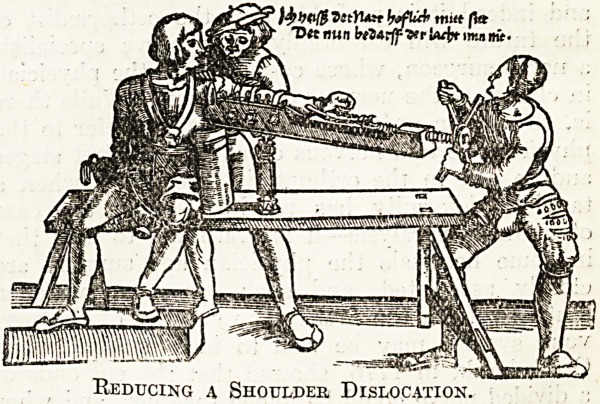# Modern Orthopædy: Recent Advances and What They Mean

**Published:** 1913-11-15

**Authors:** 


### THE ORTHOPÆDIC DEPARTMENT.

### II.—Modern Orthopædy: Recent Advances and what they Mean.

In the previous article we considered the case in favour of a general hospital having its own independent orthopædic department as a separate hospital unit. Before dealing with that unit, its planning, equipment, furnishing, staffing, and working, it may be as well briefly to review the present position of orthopædy as a science and art, and to see what advances it has made during

OLD APPARATUS FOR REDUCING DISLOCATIONS.

recent years, and what claims it may urge for its recognition as a distinct branch of surgery.

Considered merely as that department of surgery which treats of the cure and relief of bodily deformity, orthopædy is by no means new. Indeed some of the most brilliant efforts of "bloodless surgery" have been anticipated by old surgeons who had few of the means which make these operations and manipulations so relatively easy for their modern colleagues. Moreover, there are some manipulations, described, or at least suggested, by older surgeons, which no modern orthopædist has yet dared to execute. This may seem strange to some of us who are not well acquainted with the history of medicine, but it is nevertheless a fact. Thus Hippocrates suggested what is known as the intra-peritoneal method of reducing a vertebral dislocation—an operation which has never been executed on a living patient. The method consisted, according to Haeser, in opening the patient's abdomen, shoving aside the intestines, and applying pressure from within upon the displaced vertebræ, while assistants made extension and counter-extension by pulling upon the trunk. The reposition of the congenitally displaced hip was suggested by Indian surgeons, and was probably practised with some success, as it is still practised among certain savage races. Mediæval surgeons described various methods for reducing dislocations, and figured apparatus for such cases. The accompanying two woodcuts, reproduced from Gersdorf's "Feldtbuch der Wundsarztney," published at Strassburg in 1528, illustrates the mechanical ingenuity of these wonderful machines. In the first block the apparatus is designed for stretching an ankylosed knee; in the second for reducing a shoulder dislocation. With the more correct knowledge of anatomy, brought about by the study of the dissected body and the pioneering work of Vesalius, these mechanical contrivances gave place to manipulative methods of reduction, some of which are still used, as, for instance, in a reduction of a traumatic dislocation of the thigh.

But orthopædy, before the time of Lister, was largely based on rough-and-ready methods; it was "bloodless" in so far that the surgeon did not dare, in the majority of cases, to operate by open methods, since the risk of "wound fever" or sepsis was too great. This was especially the case in deformities, whose relief or cure may be considered in the nature of an operation of convenience and not of urgency. A patient with a wry-neck or a tuberculous kyphosis of the spine was swathed in bandages and immobilised, usually, in the former case at least, with little permanent benefit, although in the latter there is little doubt that the rest and absence of irritative treatment did in many cases conduce to a cure. Anyone who reads the older books on surgery and notes the profound respect with which all the ancient authorities treat a lumbar abscess, for instance, will agree that in the circumstances not much progress was possible until the field had been cleared by the researches of the Glasgow surgeon whose technique made it possible for surgeons to venture upon more drastic methods, without feeling that they were undertaking risks which the case did not justify.

With the perfection of aseptic measures the advance was rapid, although orthopædics was

REDUCING A SHOULDER DISLOCATION.

still largely in the hands of the general surgeon, who treated it as a side issue. When it was found that, with proper aseptic precautions, every variety of operation could be undertaken, certain open methods of relieving bodily deformities were evolved which, owing to various causes, were foredoomed to failure. Thus the open method of reducing a dislocated hip was, for a time, the standard method; yet its results were most discouraging so far as permanent amelioration of the patient's condition was concerned, for in the majority of cases it produced an ankylosis and not that free movement which it is the surgeon's hope to restore. In their excess of zeal to overcome all deformities by open methods of operation, surgeons lost sight of the older manipulative and so-called bloodless methods, which fell largely into the hands of quacks and bonesetters, whose results were ignored by the profession, though appreciated by the public. In the early 'eighties of the last century there was a notable revival of interest in these older methods, due in some degree to the non-success of the more radical open methods. A few years later these earlier methods were perfected by various orthopædists, who set themselves seriously to work in this special branch and devoted all their attention to the elucidation of orthopædic questions. Lorentz in Austria, Hoffa in Germany, and Calot in France were pioneers in this department, and were followed later on by Paci in Italy and Goldtwaithe in America. Much attention was concentrated on the pathology and morbid anatomy of rachitic deformities, and on lesions produced by tuberculosis, trauma, and nervous diseases. All this pioneering work was acknowledged by the profession, and in Germany by the Government in creating a special professorship of orthopædic surgery at the University of Berlin, the first holder of the chair being a colonial, Hoffa. To-day the work accomplished by these pioneers and their successors is generally recognised, and more than one university and hospital have added to their staffs specialists in orthopædics, whose teaching has produced different schools.

At the present moment it may be said that the orthopædist is concerned not primarily with deformities, but with the diseases that occasion them. This extension of his limits has brought him into the wide field of the surgery of the nervous system, and indeed it is probable that the orthopædist of the future will essentially be a nerve specialist, a nerve surgeon, whose co-partner is the physician in charge of the nervous department. While there is, as yet, an unfortunate tendency to refer to the physician certain nervous cases in their first stages and to call in the orthopædist then only when a tangible deformity has resulted—as in the case of infantile paralysis—it is gratifying to note that in some hospitals the physician and surgeon are closely associated, and such cases are watched mutually from the first. The surgery of the nervous system may be said to have started when Cruikshank, in 1776, showed that the cut ends of a divided nerve could be joined together, and when Haighton showed that, after such restoration of integrity of tissue the function of the nerve could be restored. Since then numerous surgeons and physicians have worked at the subject, and the results obtained have been most encouraging. Monkeberg and Bethe investigated the histology of the process of restoration of function; Letievant modified and perfected the surgical technique—to mention only a few names at random—while more recently Marenghi, Manasse, Despres, Dumstrey, Sick, Lange, Bardenheuer, and, above all, Spitzy have shown what success could be obtained, even in seemingly hopeless cases, by applying the methods in practice. By interrupting certain nerve currents, switching others on, and modifying the action of certain muscles, it has been made possible to restore the function of paralysed limbs as in cases of infantile paralysis, spastic diplegia, and hemiplegic deformities. These are the diseases in which the orthopædist has had the most brilliant success, but noteworthy success has also attended newer methods for the treatment of other deformities, such as wry-neck, lateral curvature of the spine, angular curvature, deformities of the feet congenital or acquired, and deformities of the spine due to malformations. It is impossible to deal with them all in detail; with some of them we shall have to deal at length in a future article, but it may be said here that much of this success has been due to a happy combination of the open method with bloodless methods and with a perfected after-treatment in which mechanical apparatus, electricity, massage, and gymnastics have been highly useful auxiliaries. There is still a certain amount of opposition between two schools—the closed method school of Lorentz and the school that favours open methods—but this antagonism is daily lessening as the indications for the employment of one or the other method are becoming better known and understood. Mention must be made also of the advance made in the treatment of lesions by means of instruments. The discovery of the usefulness of celluloid has undoubtedly been a great benefit to the orthopædist; plaster of Paris and celluloid are stand-bys which he can to-day little afford to lose. Equally useful has been the employment of massage and gymnastics, and of balneotherapy and electrical treatment.

## Figures and Tables

**Figure f1:**
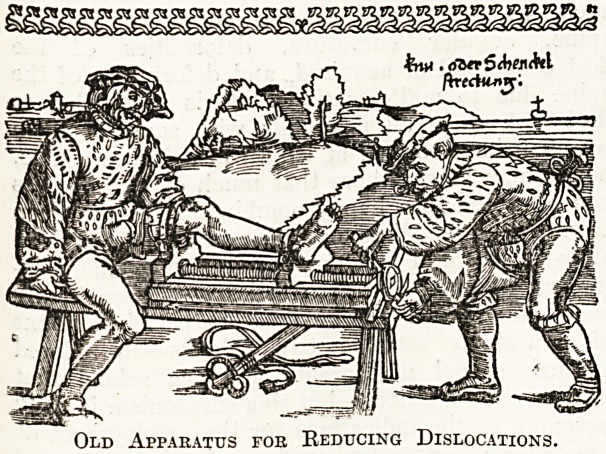


**Figure f2:**